# A new species of *Myrmozercon* Berlese (Acari, Mesostigmata, Laelapidae) associated with ant from Iran

**DOI:** 10.3897/zookeys.272.4404

**Published:** 2013-02-22

**Authors:** Azadeh Ghafarian, Omid Joharchi, Alireza Jalalizand, Mahdi Jalaeian

**Affiliations:** 1 Department of Entomology, Collage of Agriculture, Khorasgan Branch, Islamic Azad University, Isfahan, Iran; 2 Department of Plant Protection, Rice Research Institute of Iran (RRII), Rasht, Iran

**Keywords:** Laelapidae, *Myrmozercon*, ants, taxonomy, Iran, myrmecophiles, nest

## Abstract

This paper report on a new species of mites of the genus *Myrmozercon* associated with ant in Iran – *Myrmozercon cyrusi* Ghafarian and Joharchi **sp. n.** was collected associated of the *Monomorium* sp. in Kenevist Rural District in the Central District of Mashhad County, Khorasan Razavi Province, Iran. This new species is described and illustrations provided. *Myrmozercon ovatum* Karawajew, 1909 is suspected to be a junior synonym of *Myrmozercon brevipes* Berlese, 1902 and host-specificity and host range of *Myrmozercon* are also reviewed.

## Introduction

Many species Laelapidae have been reported from ants or their nests. The myrmecophiles genus *Myrmozercon* includes about 22 described species from Europe, Australia, Africa, Middle East, Transcaucasia, North America and Central Asia (Michael 1891, Hunter and Hunter 1963, Rosario and Hunter 1988, Karawajew 1909, Ueckermann and Loots 1995, Walter 2003, Shaw and Seeman 2009, Trach and Khaustov 2011, Joharchi et al. 2011).

All species are associated with ants, except for one intercepted at quarantine on plant material (Hunter and Hunter 1963). Shaw and Seeman (2009) synonymised *Parabisternalis* Ueckermann and Loots, 1995 with *Myrmozercon*, and included the subgenus *Myrmonyssus* (*Laelaspulus*) Berlese, 1904 as a synonym of *Myrmozercon*. The only species known from western Asia and Eastern Europe are *Myrmozercon ovatum* Karawajew, 1909 from Turkmenistan, *Myrmozercon tauricus* Trach & Khaustov, 2011, from Ukraine and *Myrmozercon Karajensis* Joharchi et al., 2011 from Iran. In this paper, we describe a new species of *Myrmozercon* found in Iran.

## Materials and methods

Laelapidae associated with ants were collected mainly in Khorasan Razavi Province over a period of two years. Mites were removed from ants’ nests by hand picking or by extraction from ant nesting material using Tullgren funnels. Mites were cleared in Nesbitt’s solution and mounted in Hoyer’s medium. The nomenclature used for the dorsal idiosomal chaetotaxy is that of Lindquist and Evans (1965), the leg chaetotaxy is that of Evans (1963a), the palp chaetotaxy is that of Evans (1963b), and names of other anatomical structures mostly follow Evans and Till (1979). We use the term “lyrifissures” to refer to slit-shaped sensilli, and “pore” for circular or oval-shaped cuticular openings of unspecified function. Holotype and paratypes of the new species are deposited in the Acarological collection, Department of Plant Protection, Yazd Branch, Islamic Azad University (YIAU); paratypes are also deposited in the Jalal Afshar Zoological Museum, College of Agriculture, University of Tehran, Iran (JAZM) and in the Australian National Insect Collection, CSIRO Ecosystem Sciences, Canberra, Australia (ANIC). All measurements in the descriptions are given in micrometres (µm).

### Genus Myrmozercon

Berlese

http://species-id.net/wiki/Myrmozercon

Myrmozercon Berlese, 1902: 699. Type species *Myrmozercon brevipes* Berlese, 1902, by monotypy.
Myrmonyssus Berlese, 1903: 16. Type species *Myrmonyssus diplogenius* Berlese, 1903, designated by Berlese, 1904 (synonymy by Rosario and Hunter 1988).
Myrmonyssus (*Laelaspulus*) Berlese, 1904: 437. Type species *Myrmozercon acuminatus* Berlese, 1903, by original designation (synonymy by Shaw and Seeman 2009).
Parabisternalis Ueckermann & Loots, 1995: 35. Type species *Parabisternalis yemeni* Ueckermann & Loots, 1995, by original designation (synonymy by Shaw and Seeman 2009).

#### Notes on the genus.

The diagnosis of *Myrmozercon* used here is based on that of Shaw and Seeman (2009). Most species of *Myrmozercon*, including the type species *Myrmozercon brevipes*, show moderate to strong hypertrichy on the dorsal shield. However, *Myrmozercon burwelli* Shaw & Seeman, 2009 (24-25 pairs), and the new species, have a reduced dorsal chaetotaxy. All species appear to have asymmetrical and unpaired setae on the dorsal shield, which makes it difficult to recognise their homology except the new species. In most species the dorsal shield is reduced or truncated posteriorly to expose a strip of unsclerotised opisthonotal skin, but this is not true for every species. Species of *Myrmozercon* also vary in the presence or absence of metasternal setae st4, the sternal shield of new species is extended to which the sternal shield is fused with the endopodal plates, with three pairs of setae and three pairs of lyrifissures and metasternal setae (st4) absent. The leg chaetotaxy of *Myrmozercon* species is variable, and does not provide diagnostic characters that define the genus (Shaw and Seeman 2009) and this is very characteristic and fixed in the new species. The new species has one ventral seta on the palp trochanter the same as in most species of *Myrmozercon*. Shaw and Seeman (2009) described a swelling on the dorso-distal edge of the palp trochanter in several species, but this structure is not present in new species. This instability in morphology, and the edentate chelicerae and short peritremes of *Myrmozercon*, suggest that *Myrmozercon* is parasitic on its ant hosts, and not simply a commensal in its host’s nests, but this has not been established experimentally. The specimens of new specieswere found clinging to the abdomen and head of the ants.

## Results

### Myrmozercon cyrusi

Ghafarian & Joharchi sp. n.

urn:lsid:zoobank.org:act:5DD70D4C-1312-49FA-A881-C663EB04E98E

http://species-id.net/wiki/Myrmozercon_cyrusi

Figures 1–13

#### Type material.

Holotype, female, Kenevist Rural District in the Central District of Mashhad County, Khorasan Razavi Province, Iran, 36.97'N, 59.68'E, alt. 945 m, 25 April 2012, A. Ghafarian coll., in nest of *Monomorium* sp. (in YIAU). Paratypes, four females, same data as holotype (in JAZM and ANIC).

#### Description of the female.

**Figures 1–13.**
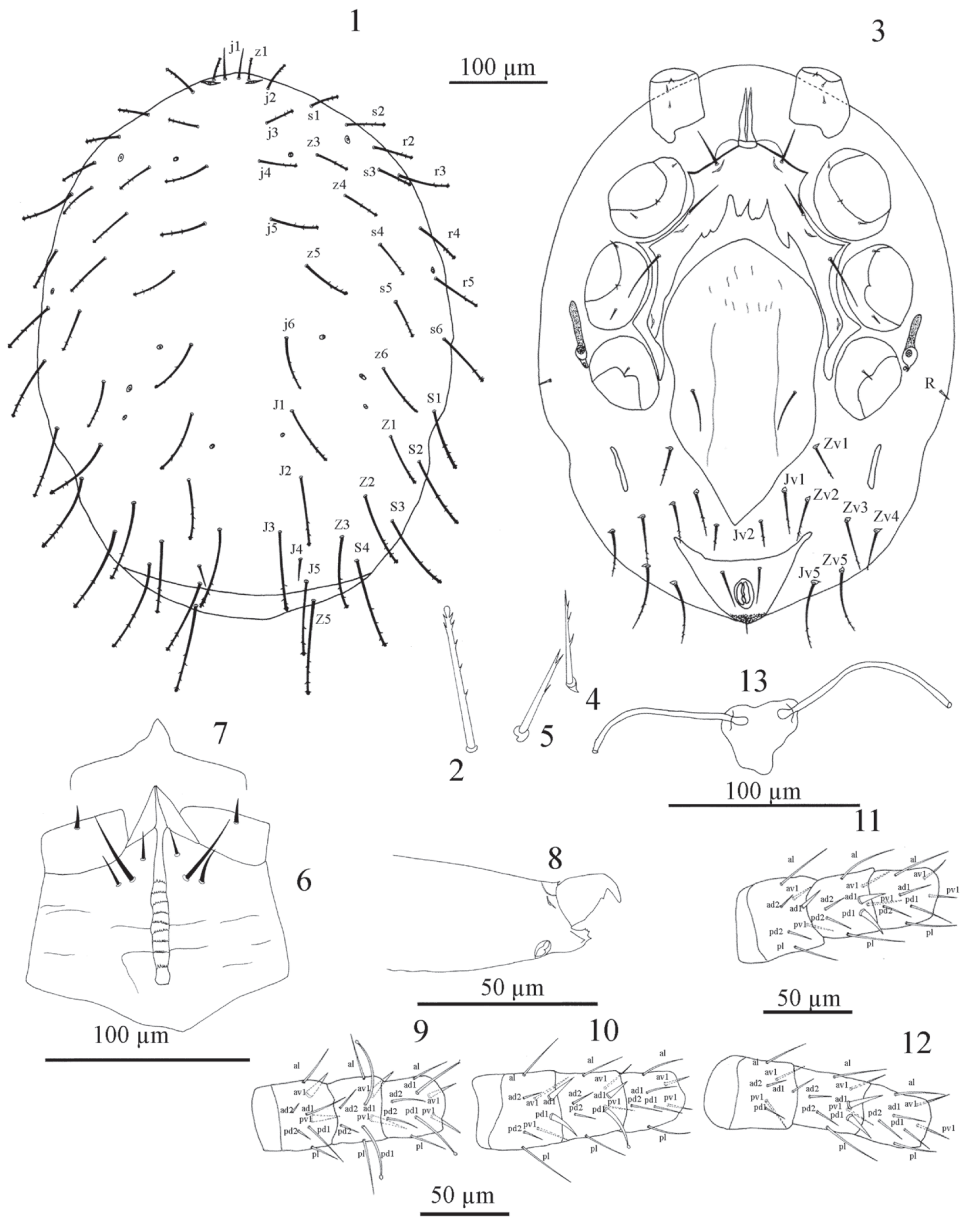
*Myrmozercon cyrusi* Ghafarian and Joharchi sp. n., female. **1** Dorsal shield **2** Dorsal seta enlarged (J5) (not to scale) **3** Ventral idiosoma **4–5** Opisthogastric setae enlarged (not to scale) **6** Hypostome **7** Epistome **8** Chelicera **9** femur, genu and tibia I, dorsal aspect **10** femur, genu and tibia II, dorsal aspect **11** femur, genu and tibia III, dorsal aspect **12** femur, genu and tibia IV, dorsal aspect **13** Insemination structures.

Figures 1–13. *Dorsal idiosoma* (Fig. 1). Length 522–534. Dorsal shield length 488–500, width 420–436 (n = 5). Shield posteriorly truncate, not covering entire idiosoma, leaving a curved strip of unprotected skin posterior to setae J5, shield without distinct reticulate ornamentation over whole surface; with 33 pairs of setae, 21 podonotal (z2 absent), 12 opisthonotal (Z4, S5 absent) and Z5 in soft skin posterior to shield, almost all setae except j1 and J4 slightly barbed in apical third or less, with club-like tip (Fig. 2), opisthonotal setae very long, reaching well past base of next posterior setae, dorsal shield setae increasing in length from anterior to posterior (j1 25-27, J1 54-59, J2 67-69, J3 79-82), without unpaired and asymmetrical seta, setae on shield uniform in length and thickness except j1 (25-27) and J4 (20-25) very fine and minute. A pair of very fine and minute setae in R series on the lateral soft skin but appear on ventral view. Shield with eight pairs of minute pores and lyrifissures including a pair of lyrifissures situated near z1, other pores inconspicuous.

*Ventral idiosoma* (Fig. 3). Tritosternum with short broad base (10-11 × 15-17 wide) fused to sternal shield, bifurcated at a short distance above suture, laciniae 37-40 in length, with smooth edges, strap-like and broad at base; pre-sternal shields fused with sternal shield. Sternal shield (length 248-255) narrowest between coxae II (104-108) widest between coxae II and III (218-22), with biconvex anterior margin and extending beyond level of st1, lateral margins thickened and posterolateral corners fused with endopodal shield; posterior margin concave; shield bearing three pairs of smooth pointed setae (*st*1 30-37, *st*2 40-45, *st*3 50-51) and two pairs of lyrifissures, one pair between setae *st*1 and *st*2 and the other between *st*2 and *st*3; surface with indistinct reticulate ornamentation. Seta *st*4 absent, metasternal pores also on extent of sternal shield but metasternal plates apparently absent. Genito-ventral shield wide, strongly tapering posteriorly, 320–346 long, 168–174 maximum width. Surface of shield smooth with longitudinal markings in anterior half; with one pair of simple setae *st*5 (35–37). Anal shield triangular, its anterior without lineate ornamentation, cribrum small, anal pores indistinct, bearing short post-anal seta 15-17 long, and a pair of para-anal setae 37–42 long. Opisthogastric skin with long, narrow metapodal plates (40–44 × 8–10 wide) and eight pairs of setae, almost all setae slightly barbed in apical third or less, each arising on small sclerotised platelet (Figs. 4, 5), (Jv1 47-50, Jv2 35-37, Jv5 64-73, Zv1 37-42, Zv2 45-50, Zv3 55-63, Zv4 45-50, Zv5 67-75). Peritreme very short (35-40), extending to posterior level of coxae Ш. Peritrematal shields absent, post-stigmatal section conspicuous, with one pair of pore.

*Gnathosoma*. Hypostomal groove with nine rows of denticles, 10 to 15 very fine denticles per row (Fig. 6). Hypostome with three pairs of setae, internal posterior hypostomal setae h3 longest, palp coxal setae absent; surface of hypostome ornamented with transverse and curved lines. Palp chaetotaxy: trochanter 1, femur 5, genu 5, tibia 12; all palp setae pointed, palp tarsal claw two-tined, dorsodistal edge of palp femur without swelling. Epistome triangular, smooth, with pointed apex (Fig. 7). Chelicera hyaline, fixed digit of chelicera reduced, with four minute terminal denticles, pilus dentilis, dorsal lyrifissure present (Fig. 8); movable digit weakly sclerotised, distally curved, with one small subterminal tooth and one stronger terminal tooth, cheliceral seta absent, arthrodial corona with hyaline flap without filaments (Fig. 8). Corniculi long, weakly sclerotised.

*Legs*: Legs II and III short (258–268, 268–272), I and IV longer (288-298). Chaetotaxy: Leg I: coxa 0 0/1 0/1 0, trochanter 1 0/1 1/1 1 (*pd* thick), femur 1 2/1 2/1 1 (*ad*1 and *pd*1 long, ventral setae all thick, Fig. 9), genu 1 2/1 2/1 1 (*ad*1 and *pd*1 long with club-like tip, ventral setae all thick, Fig. 9), tibia 1 2/1 2/1 1 (*ad*1 and *pd*1 long with club-like tip, ventral setae all thick, Fig. 9). Leg II: coxa 0 0/1 0/1 0, trochanter 1 0/1 0/2 1, femur 1 2/1 2/1 1 (*ad*1 and *pd*1 thick, *al* and *pl* long, Fig. 10), genu 1 2/1 2/1 1 (*ad*1 thick, *pd*1 long with club-like tip, *al* and *pl* long, Fig. 10), tibia 1 1/1 2/1 1 (*al* and *pl* long, Fig. 10). Leg III: coxa 0 0/1 0/1 0, trochanter 1 0/1 0/1 1 (*al* thick), femur 1 2/1 1/1 1 (*ad*1 thick, *al* and *pl* long, Fig. 11), genu 1 2/1 2/1 1 (*ad*1 and *pd*1 thick, *ad*1 in two paratypes club-like tip, *al* and *pl* long, Fig. 11), tibia 1 1/1 2/1 1 (*al* and *pl* long, Fig. 11). Leg IV: coxa 0 0/1 0/0 0, trochanter 1 1/2 0/1 0 (*ad* thick), femur 1 2/1 1/1 0 (*al* long Fig. 12), genu 1 2/1 2/1 1 (*ad*1 and *pd*1 thick, *ad*1 in two paratypes club-like tip, *al* and *pl* long, Fig. 12), tibia 1 1/1 2/1 1 (*al* and *pl* long, Fig. 12). Tarsi I-IV with 16 setae, pre-tarsi with membranous ambulacrum, claws absent.

*Genital structures*: Insemination ducts opening on posterior margin of coxa III; sacculus an irregular, dark coloured mass behind coxae IV, ducts entering sacculus via a pair of circularopenings (Fig. 13).

#### Etymology.

The species is named in memory of Cyrus the Great (Old Persian: *Kuruš*; c. 600 BC or 576 BC–530 BC) was the first Achaemenian Emperor of Persia, as the “father of the Iranian nation”, who issued a decree on his aims and policies, later hailed as his charter of the rights of nations.

#### Notes.

*Myrmozercon cyrusi* differs from all other species in the genus by its very short peritreme, palp coxal setae absent, genua I-IV with similar chaetotaxy (1 2/1 2/1 1) and trochanter of palp with only one ventral seta.

## Discussion

Only eleven species of *Myrmozercon* have been described from the Palaearctic Region (*Myrmozercon acuminatus* (Berlese, 1903) on *Messor capitatus* (Latereille, 1798) from Italy; *Myrmozercon antennophoroides* (Berlese, 1904) on *Camponotus aethiops* (Latereille, 1798) from Italy; *Myrmozercon brachiatus* (Berlese, 1903) on *Messor capitatus* from Italy; *Myrmozercon brevipes* Berlese, 1902 on *Tapinoma erraticum* (Latereille, 1798) from Italy; *Myrmozercon clarus* (Hunter and Hunter, 1963) on *Crematogaster clara* Mayr from Georgia; *Myrmozercon diplogenius* (Berlese, 1903) on *Camponotus aethiops* from Italy; *Myrmozercon flexuosa* (Michael, 1891) on *Camponotus herculeanus* (L., 1758); *Myrmozercon Karajensis* Joharchi et al., 2011 on *Camponotus* sp. from Iran; *Myrmozercon liguricus* Vitzthum, 1930 on *Crematogaster scutellaris* (Olivier, 1792) from Germany; *Myrmozercon ovatum* Karawajew, 1909 one from a worker *Myrmecocystus emeryi* Karawajew, 1909, but mostly on workers of *Tapinoma erraticum* nigerrimum from Turkmenistan; *Myrmozercon tauricus* Trach & Khaustov, 2011 on *Crematogaster schmidti* (Mayr, 1853) from Ukraine). Three subfamilies and seven genera of ants have been reported as hosts from the world: Formicinae, *Camponotus*, *Cataglyphis*, *Polyrhachis*; Dolochoderine, *Iridomyrmex*, *Tapinoma*; Myrmecinae, *Crematogaster*, *Messor*. *Myrmozercon cyrusi* has been collected in association with *Monomorium* sp. and this is the first record of ant host.

According topublications, *Myrmozercon ovatum* Karawajew, 1909 shares many compelling characters with *Myrmozercon brevipes* Berlese, 1902 especially form of genital shield, short peritreme, short legs, dorsal shield highly hypertrichous and collecting on same host, but we have not had the opportunity to examine type specimens of these two species therefore we consider the *Myrmozercon ovatum* to be a suspected synonym of *Myrmozercon brevipes*.

The biology of *Myrmozercon* species has not been studied yet. However,instability in morphology, the edentate chelicerae and short peritremes might suggest that *Myrmozeron* is parasitic on its ant hosts, and not simply a commensal in its host’s nests, but this has not been established experimentally.

## Supplementary Material

XML Treatment for Myrmozercon

XML Treatment for Myrmozercon cyrusi
